# Digital Dental Triage and Access to Care: A Qualitative Study of Experiences in a Teaching Hospital

**DOI:** 10.3390/dj14070423

**Published:** 2026-07-09

**Authors:** Alaa Husni Qari, Ola Abdullah Sheiko, Mohammed Saed Althagafi, Wael Yaghmoor, Rayan Sharka

**Affiliations:** 1 Preventive Dentistry Department, Faculty of Dental Medicine, Umm Al-Qura University, Makkah 24382, Saudi Arabia; 2 Teaching Dental Hospital, Faculty of Dental Medicine, Umm Al-Qura University, Makkah 24382, Saudi Arabia; 3 Basic & Clinical Oral Sciences Department, Faculty of Dental Medicine, Umm Al-Qura University, Makkah 24382, Saudi Arabia; 4 Oral and Maxillofacial Surgery Department, Faculty of Dental Medicine, Umm Al-Qura University, Makkah 24382, Saudi Arabia

**Keywords:** digital dental triage, teledentistry, dental public health, health communication

## Abstract

**Background/Objectives**: Digital triage systems are increasingly used in dentistry to support patient access and improve service efficiency, particularly in academic and public healthcare settings. However, limited evidence exists on how these systems function in practice from the perspectives of both patients and providers. This study aimed to explore patients’ and staff members’ experiences, perceptions, and expectations of a digital dental triage system at a teaching dental hospital. **Methods**: A qualitative descriptive study was conducted at the Dental Teaching Hospital of Umm Al-Qura University (DTH-UQU), Saudi Arabia. Semi-structured interviews were carried out with 25 participants, including dental professionals, administrative staff, and patients who had recently used the digital triage system. Interviews were audio-recorded, transcribed verbatim, and analyzed using Braun and Clarke’s thematic analysis approach. **Results**: Analysis identified six interconnected themes. Patients generally found the system easy to use and consistent with their digital habits, yet this usability did not translate into satisfaction due to limited communication, post-registration silence, and unclear care pathways. Staff emphasized clinical accuracy, educational requirements, and capacity constraints as key drivers of triage decisions that were often invisible to patients. Communication breakdowns, misclassification, duplicate registrations, and unmet expectations emerged as central challenges, contributing to workflow inefficiencies. Participants suggested that improvements focused on enhanced communication, guided data entry, system integration, and expanded teledentistry functionality. **Conclusions**: The effectiveness of digital dental triage systems depends not only on usability but also on transparent communication, alignment with institutional capacity, and integration within broader care pathways.

## 1. Introduction

The quality of healthcare delivery is shaped not only by clinical skills and treatment protocols, but also by how effectively patients are guided into appropriate care [1]. From a dental public health perspective, triage systems play a critical role in managing access, prioritizing need, and ensuring that limited clinical resources are used efficiently [2]. Structured triage systems have therefore been adopted in both emergency medicine and dentistry to manage patient flow, stratify cases by urgency, and direct patients to suitable care pathways [3,4,5]. When integrated with standardized care pathways and digital decision support tools, these systems can streamline workflows, reduce avoidable visits, and improve patient experience at the population level [6,7,8]. Well-designed triage processes also help shorten waiting times and optimize the use of available healthcare resources, contributing to more equitable and efficient service delivery [4,8,9].

A systematic review by Farzandipour et al. (2024) reported that structured tele-triage can reduce unnecessary emergency department visits by 1.2% to 22.2%, while also improving patient satisfaction, with reported rates ranging from 53% to 98% across diverse settings [9]. In dentistry, the accuracy and timeliness of triage decisions are particularly important, as delays or misclassification can exacerbate oral disease and increase demand for urgent care [1]. During the COVID-19 pandemic, telephone-based dental triage enabled the remote management of urgent dental complaints, with studies indicating that approximately 49–70% of cases could be safely managed without face-to-face visits [3,4]. During periods of high demand, dental triage systems also support symptom control and preserve in-person clinical capacity for patients with time-sensitive needs, reinforcing their value as a public health tool [3,4].

Beyond traditional telephone triage, recent technological advances including teledentistry platforms, mobile health (mHealth) applications, and AI-assisted systems have expanded the scope and precision of remote patient assessment and care routing [7,10,11]. These technologies typically incorporate structured symptom questionnaires, image capture, and real-time communication features, which together support more responsive and accurate triage decisions [7,10,12]. Studies suggest that AI-supported and smartphone-based triage systems can improve operational efficiency and patient experience [7,11]. From a dental public health standpoint, such tools have the potential to reduce waiting times and improve access to care, although evidence remains variable across clinical contexts and implementation models [7,10,11]. A systematic review by Gurgel-Juarez et al. (2022) reported that teledentistry can improve access and demonstrates a diagnostic sensitivity of 80–88% and specificity of 73–95% for referral and treatment planning, with performance influenced by modality and setting [10].

Despite the expanding adoption of digital triage solutions, persistent systemic and operational inefficiencies continue to limit their public health impact. These challenges can undermine triage effectiveness by delaying access to care and negatively shaping patient experience [6,13]. Structural barriers including limited digital infrastructure, workforce shortages, and socioeconomic constraints further restrict equitable access and influence care-seeking behavior [6,14]. Such issues are particularly evident in Saudi public hospitals, where resource imbalances, workforce limitations, and fragmented digital systems contribute to inefficiencies and delays in service delivery [15,16]. In these contexts, unclear or poorly integrated triage pathways may result in patients being directed to inappropriate clinics, disrupting continuity of care and diminishing overall system performance [11,17].

Within academic dental institutions, triage systems play a dual role: facilitating access to care while aligning case complexity with the educational requirements of students and interns. At the Dental Teaching Hospital of Umm Al-Qura University (DTH-UQU), patients begin their care journey by completing a digital triage form that captures demographic information and chief complaints prior to formal registration. However, reliance on self-reported symptoms can limit diagnostic accuracy, as patients may not fully capture the clinical context of their condition. This can lead to inconsistencies and potential misclassification [14,18]. Existing evidence shows wide variation in self-triage accuracy, ranging from 11.5% to 90.0%, underscoring the need for careful validation of automated triage tools to ensure patient safety and appropriate resource allocation [14,18].

Following submission, triage staff review patient information and assign cases to appropriate clinics or student providers. In practice, this process is often constrained by misalignment between patient needs and educational requirements, repeated registrations, and unclear prioritization criteria. These challenges can create confusion, prolong waiting times, and delay access to care, with implications not only for individual patients but also for system efficiency and equity [16].

To date, much of the research on triage systems has focused on technical performance indicators, such as diagnostic or triage accuracy, which vary widely across systems and settings [14]. In contrast, patient-centered outcomes, particularly user experience and patterns of service use, have received less attention [6]. This limits our understanding of how triage system design influences patient behavior, continuity of care, and psychological responses such as anxiety or uncertainty. Emerging evidence highlights that patient experience, shaped by communication quality and system usability, is a key driver of satisfaction and continued engagement with digital health services [7,19]. However, many studies remain focused on narrow measures, such as satisfaction scores, with limited exploration of broader experiential and contextual factors that influence engagement and access [7,19].

Despite the growing reliance on digital triage systems, qualitative research examining the experiences of patients and healthcare staff particularly within teaching hospitals and Middle Eastern contexts remains scarce [20]. From a dental public health perspective, understanding these experiences is essential to ensure that digital triage systems are usable, equitable, and responsive to population needs [7]. These gaps highlight the importance of examining how patients perceive triage systems and how such perceptions shape adherence, trust, and subsequent care-seeking behavior.

Accordingly, this qualitative study aims to explore the perceptions, experiences, and expectations of patients and staff regarding the digital triage system at DTH-UQU. By identifying communication gaps and usability challenges, this study seeks to amplify the voices of service users and providers and to inform the development of patient-centered, efficient triage processes that support clinical education while advancing equitable access to oral healthcare.

## 2. Materials and Methods

### 2.1. Ethical Considerations

Ethical approval for this study was obtained from the Institutional Review Board of Umm Al-Qura University (Approval No. HAPO-02-K-012-2024-02-1985, on 13 February 2024). All participants were provided with written information about the study and gave informed consent prior to participation. Consent was obtained before each interview, and participants were assured of confidentiality and their right to withdraw at any stage without consequence.

### 2.2. Study Design

This study employed a qualitative descriptive design to explore the perspectives of dental professionals, administrative staff, and patients regarding the current electronic triage system at DTH-UQU. The study was conducted and reported in accordance with the Consolidated Criteria for Reporting Qualitative Research (COREQ) guidelines (Supplementary File S1).

### 2.3. Setting and Sample

The study was conducted at DTH-UQU, an academic dental institution located in Makkah, Saudi Arabia. A purposive sampling strategy was used to recruit a diverse group of participants from three key stakeholder categories: dental professionals representing major clinical specialties, administrative staff involved directly in the triage process, and patients who had recently used the electronic triage system.

Dental professionals and administrative staff were selected based on their seniority and active involvement in patient triage procedures. Staff participants were eligible if they were currently affiliated with DTH-UQU and had at least two years of professional experience. Patient participants were eligible if they were aged 18 years or older and had completed the electronic triage process at DTH-UQU. Individuals not affiliated with DTH-UQU or with less than two years of professional experience were excluded. Patients were identified from records of individuals who had used the electronic triage system during the preceding six months. Eligible patients were contacted, provided with an information sheet and consent form, and invited to participate in the study. A total of 20 patients were invited, of whom 16 agreed to participate.

Dental professionals and administrative staff were invited to participate via institutional email. A total of 12 staff members were invited, of whom 9 agreed to participate. For those who expressed interest, interviews were scheduled at a convenient time. Patient interviews were conducted by telephone, while interviews with dental professionals were carried out in their offices.

### 2.4. Data Collection

Data were collected between 1 September 2024 and October 2025 through semi-structured, individual interviews conducted by O.S. and M.A., both qualified dentists.

A topic guide, informed by the existing literature and reviewed by subject-matter experts, was used to support consistency across interviews (Table 1) [1,14,16]. Patient interviews were conducted in Arabic to allow participants to express their experiences comfortably in their native language. All interviews were audio-recorded and transcribed verbatim in Arabic. The transcripts were then translated into English by bilingual members of the research team who were proficient in both languages and familiar with the research context. To ensure translation quality and preserve meaning, the translated transcripts were reviewed against the original Arabic versions by a second bilingual researcher. Any discrepancies or ambiguities were discussed within the research team and resolved by consensus. In addition, a purposive subset of transcripts was independently checked to further support the accuracy and consistency of the translation. Staff and faculty interviews were conducted in English. Translations were reviewed within the research team to ensure accuracy and preservation of meaning.

Two pilot interviews with patient participants were conducted by both researchers to assess the clarity and suitability of the interview questions, ensure consistent use of the topic guide, and minimize potential interviewer bias [21]. Data from the pilot interviews were not included in the final analysis.

To maintain consistency across interview modes, all interviews followed a semi-structured format. Interviewers used similar probing and rapport-building approaches to encourage detailed responses. In telephone interviews, additional attention was paid to verbal cues, such as tone, pauses, and emphasis, to account for the absence of non-verbal communication. These measures were intended to support both the depth and comparability of data across formats.

The interview guide consisted of open-ended questions exploring participants’ experiences with the electronic triage system, perceived effectiveness and outcomes, expectations, and suggestions for improvement. Interviews typically lasted between 15 and 45 min. NVivo 14 software (Lumivero, Denver, CO, USA) was used to support data organization, management, and coding.

### 2.5. Data Analysis

All audio recordings were transcribed verbatim before analysis. The data were analyzed using Braun and Clarke’s (2006) reflexive thematic analysis approach [22], following its six phases: (1) familiarization with the data, (2) generation of initial codes, (3) searching for themes, (4) reviewing themes, (5) defining and naming themes, and (6) producing the final report.

The analysis followed a primarily inductive approach, allowing themes to emerge from the data rather than being driven by pre-existing theoretical frameworks. However, the process was also informed by the study aims and relevant research, reflecting a more pragmatic and exploratory orientation.

NVivo 14 software was used to support data management, coding, and organization. It also facilitated comparisons across participant groups and provided a space for storing analytic notes and documenting emerging patterns during the analysis.

A coding framework was developed iteratively by the research team and refined as the analysis progressed. Initial coding was conducted independently by members of the team to support close engagement with the data. Codes and emerging themes were then compared and discussed in regular team meetings, where differences in interpretation were examined and used to refine code definitions and strengthen the analysis. This iterative process helped establish a shared understanding of the coding framework.

Decisions related to code development, refinement, and theme generation were documented throughout, creating an audit trail to enhance transparency and rigor. To further support trustworthiness, member checking was conducted with a purposive subset of participants (n = 8), including both patients and staff. Participants were contacted following preliminary analysis and provided with a summary of the key findings. They were invited to comment on whether the interpretations reflected their experiences [23]. No major discrepancies were identified during this process.

Data collection and analysis were conducted concurrently and continued until thematic saturation was reached, defined as the point at which no new codes, categories, or themes emerged from successive interviews. Saturation was assessed iteratively, and recruitment was discontinued once additional interviews yielded no substantially new insights [24].

To ensure trustworthiness, this study addressed credibility, dependability, confirmability, and transferability. Credibility was supported through prolonged engagement with the data, iterative analysis, and member checking with participants. Dependability was enhanced by maintaining a clear audit trail documenting coding decisions, theme development, and analytic processes. Confirmability was supported through reflexive practices and team discussions, ensuring that interpretations were grounded in the data rather than researcher assumptions. Transferability was facilitated by providing a detailed description of the study context, participant characteristics, and data collection processes, enabling readers to assess the applicability of findings to similar settings [25].

### 2.6. Methodological Rigor and Reflexivity

Rigor and trustworthiness were addressed throughout the study in accordance with established qualitative research principles. Credibility was enhanced through the use of semi-structured interviews, iterative data analysis, and member checking with selected patient and staff participants to ensure that interpretations accurately reflected their experiences. Confirmability was strengthened through independent coding by multiple researchers and regular analytic discussions to compare interpretations and resolve discrepancies by consensus. Transferability was facilitated by providing a detailed description of the study setting, participant characteristics, and research context.

Reflexivity was considered throughout the research process. The interviews were conducted by O.S. and M.A., both qualified dentists with academic and clinical backgrounds in dental and health sciences and formal training in qualitative research methods. Both interviewers graduated from the same dental school and were familiar with the institutional context of the study site. No prior personal relationship was established with patient participants before study commencement. Participants were informed about the purpose of the study, the researchers’ professional roles, and the aim of exploring experiences with the digital triage system to inform service improvement.

The researchers maintained reflexive awareness throughout data collection and analysis. During interviews, they relied on open-ended questions and avoided leading prompts, allowing participants to describe their experiences in their own terms. For example, rather than assuming a shared understanding of clinical procedures, interviewers encouraged participants to explain their experiences in detail, even when these appeared familiar.

The researchers also considered how their professional backgrounds might shape interpretation. During analysis, regular discussions were used to revisit initial assumptions and explore alternative readings of the data. Instances in which participants’ accounts diverged from expected clinical pathways were examined closely rather than set aside, allowing for the development of new insights.

At the same time, the team’s clinical and institutional familiarity informed the interpretation of nuanced responses, particularly in relation to workflow challenges, patient–provider interactions, and system constraints within the teaching hospital context. This combination of reflexive awareness and contextual understanding supported both the depth and credibility of the analysis.

## 3. Results

A total of 25 interviews were conducted, at which point data saturation was reached.

### 3.1. Participant Characteristics

The patient sample comprised nine females and seven males, with ages ranging from 19 to 49 years. Participants had varied educational backgrounds, including intermediate school, high school completion, and current university study. Employment status also differed across the sample and included students, individuals working in professional roles, and those not currently employed, such as homemakers. This diversity allowed for a wide range of perspectives on experiences with the digital dental triage system.

The study also included nine staff and faculty participants representing a range of dental specialties and leadership roles within the teaching hospital. These participants included clinicians and academics from operative dentistry, endodontics, orthodontics, periodontology, oral surgery, and prosthodontics, in addition to senior academic and administrative leadership.

The staff group consisted of five females and four males, aged between 38 and 60 years. Participants brought extensive clinical, teaching, and administrative experience and were directly involved in patient care, education, and service management. This breadth of experience enabled insights into the digital triage system from clinical, educational, and operational perspectives.

### 3.2. Emeregent Themes

Analysis of the interview data resulted in six themes that capture how patients and staff perceive, experience, and evaluate the electronic triage system at DTH-UQU.

These themes and their corresponding subthemes are presented in Table 2. The subsequent paragraphs provide excerpts from participants’ interviews.

#### 3.2.1. Theme 1: Interface Usability Versus Clinical Needs

Both patients and staff engaged positively with the electronic triage system; however, a clear tension emerged between patient-valued simplicity and staff-required clinical accuracy.

##### Subtheme 1: Patients Prioritize Usability and Accessibility

Patients consistently described the triage link as easy to navigate, intuitive, and compatible with their everyday digital practices. Many emphasized that the process felt straightforward and required little effort. As one patient explained, *“Very easy, I just filled in the required information” (P1)*, while another stated that *“Nowadays everything is electronic, so it was simple and straightforward” (P16)*. For patients, this ease of use was closely associated with perceived system quality and initial satisfaction.

##### Subtheme 2: Staff Concerns About Insufficient Clinical Detail and Misclassification

In contrast, staff viewed this simplicity as a limitation. They noted that patient self-reported information often lacked sufficient clinical detail, leading to misclassification. As one faculty member explained that *“The form is as basic as possible. The patient cannot guide himself to reach the correct triage, he will just put anything” (F2)*. Another noted that *“The patient cannot differentiate between endodontic and operative cases, even pain is not an indicator” (F1)*.

These limitations had direct workflow consequences. When patients were assigned to inappropriate clinics, they were often redirected and asked to re-register. One clinician described this cycle: *“When the doctor sees it’s the wrong case, he sends him back to the link again… it becomes a closed circle” (S3)*. Patients also recognized these gaps when preset options failed to capture their condition: *“My problem was oral diseases, and there was no clear option” (P12)*.

#### 3.2.2. Theme 2: Communication Breakdown as the Core System Failure

Communication problems were a consistent issue across both the patient journey and internal workflows, contributing to dissatisfaction and inefficiency. Participants described delays in communication after registration, weak coordination between teams, and a lack of clear expectation setting. As a result, patients often felt left in the dark, while staff found themselves responding to ongoing complaints, both within the hospital and in public spaces.

##### Subtheme 1: Post-Registration Silence for Patients

Patients described the system as a “black box” after submission, with no acknowledgment or follow-up. Many reported repeated submissions due to lack of response. As one patient stated that *“Nothing happened. No one contacted me” (P13)*. The lack of feedback often led patients to resubmit the form repeatedly in an attempt to prompt a response, with one participant reporting, *“Maybe 10 times or 5 times, and still no reply” (P16)*. This uncertainty appeared to intensify frustration and likely contributed to the creation of duplicate records.

Several participants also explained that the absence of follow-up communication made it difficult to recall what steps, if any, they were expected to take next. As one patient reflected, *“After that? I don’t remember” (P1)*. Waiting without a clear timeframe further compounded this experience. Participants spoke of prolonged periods of uncertainty, such as, *“Until now, no one has contacted me” (P16)*. Even patients who otherwise viewed the system as easy to use identified communication as its main limitation. One participant noted that *“The only downside is that I still haven’t received an appointment, it takes a long time from the hospital” (P1)*.

##### Subtheme 2: Internal Coordination Gaps and Public Complaint Exposure

Staff accounts consistently pointed to gaps in coordination between departments, students, and administrative teams, noting that these internal inconsistencies often become visible to the public through patient complaints. A senior administrator stressed the importance of shared responsibility, stating that *“Cooperation is essential. No single person can make it succeed. Sometimes students bring cases but do not properly enter information, and this creates confusion” (S1)*. Participants also reflected on how patient-facing communication breaks down when clinical terminology is not translated into accessible language. As one faculty member explained, *“Patients with lower education will say I have a hole or a cavity… patients won’t really understand technical terms” (F1)*.

This awareness was frequently accompanied by practical suggestions aimed at improving communication rather than fundamentally redesigning the system. One participant suggested simplifying the language, noting that *“You can make the questions more simple, a checklist, for example” (F1)*, while another highlighted literacy barriers: *“Some patients cannot read or write, it may help if a designated person or the student fills the form for the patient” (S1)*.

Importantly, staff were highly aware of public scrutiny and reputational risk, with one participant noting that *“We have seen this in Google Maps comments. This happens because of this problem” (S1)*.

#### 3.2.3. Theme 3: Hidden Organizational and Educational Constraints

This theme highlights a gap between how staff and patients understand the teaching hospital system. Staff work within constraints related to training, student skill levels, and clinic capacity, while patients are generally unaware of these internal processes.

##### Subtheme 1: Educational Matching Is Necessary but Invisible

Staff consistently emphasized that, as a teaching institution, matching case complexity to student level is non-negotiable. An operative faculty member explained that *“Fourth-year students are at a fundamental level. We make sure they get the simplest cases, like minimal preparation. We don’t give them complex surfaces or deep caries with possible pulp exposure” (F1)*. As students’ progress, case flexibility increases: *“For fifth year, they treat the patient as a whole, they can work with anything,”* while *“sixth-year students receive advanced aesthetic cases and indirect restorations” (F1)*. Interns, in contrast, were described as functioning *“as a complete clinic,” (S1)* without restrictions based on case suitability.

However, patients are not aware of this process and may see reassignment as inconsistency rather than a requirement. As one staff observed, *“Senior students have more flexibility. Lower-level students struggle due to their requirements, and this difficulty continues as long as the same mechanism is used” (S1)*.

The educational model also shaped daily clinic workflow. Faculty described how on-the-spot screening consumed substantial student time, particularly for junior students with limited clinical sessions. One faculty noted that *“Sometimes students do screening on the spot, waiting for screening and opening the file in the same session” (F1)*. This burden was most evident among fourth-year students: *“It takes time, especially for fourth years—they want the patient for one or two sessions” (F1)*. Staff also highlighted the impact on patients, explaining that immediate screening could result in wasted visits when cases were ultimately deemed unsuitable, *“wasting the patient’s time and the student’s time” (F2)*.

##### Subtheme 2: Patient Expectations of Universal Eligibility

In contrast, patients often assumed that completing the digital triage process would lead directly to treatment. Many expected to be placed on a waiting list, contacted quickly, and scheduled for care. When this did not happen, it contributed to confusion and unmet expectations. As one patient explained, *“I expected it to arrange things and place me in the waiting list” (P16)*. Others anticipated prompt clinical follow-up, such as receiving *“an appointment so I could come for a check-up and get a treatment plan” (P1)*. Several patients also expected rapid communication after submission, expressing views such as *“I expected they would contact me soon,” (P3) or “I thought I would get an appointment quickly.” (P14)*.

##### Subtheme 3: Structurally Determined Dissatisfaction

This gap in understanding between staff working within a teaching-based system and patients expecting universal eligibility often led to frustration. Staff recognized that, due to training requirements and limited capacity, not all cases could be treated, even with improved access through digital triage. As one staff noted, *“It is not possible to complete all cases, there are issues from registration until treatment” (S1)*. While the triage system expanded intake, it did not increase clinical capacity, student availability, or treatment quotas.

#### 3.2.4. Theme 4: Operational Consequences of Capacity Mismatch

Both patients and staff experienced delays and inefficiencies along the triage-to-treatment pathway, though their perspectives differed. Staff pointed to system bottlenecks, limited capacity, and the burden of duplicate registrations, while patients described long and uncertain waits that often led them to seek help in person.

##### Subtheme 1: Workflow Bottlenecks Consume Clinical Time

Staff consistently described the impact of same-day processes such as screening, file opening, panoramic radiographs on clinical productivity, particularly for junior students whose sessions were short and instructionally dense. Faculty noted that students often spent a significant portion of their clinical session preparing patients rather than treating them. One operative faculty member explained that *“Sometimes they do screening on the spot, which takes time… the student has a session from 9 to 12, and they wait for screening and opening the file with the panorama at the same session.” (F1)*. These delays disproportionately affected fourth-year students: *“That takes time, especially if they are fourth year, they want the patient for one session or two, maybe.” (F1)*. The implications also extended to patients, who could be screened and then found ineligible for treatment at that time, leading to what one clinician described as *“wasting the patient’s time and the student’s time.” (F3)*.

##### Subtheme 2: Demand–Supply Imbalance and Duplicate Registrations

Staff highlighted the scale of the demand–supply mismatch as a central challenge. The digital link facilitated broad access, but the number of registrations rapidly exceeded the capacity of available students and clinic slots. As the director explained, *“We have a very large list and registration is still open, many of the cases students mark as ‘consultation and assessment’ are not taken by students, so those patients keep coming.” (S1)*. Despite the digital system enabling more efficient intake than paper-based methods, staff acknowledged that *“it is not possible to complete all cases.” (S3)*.

Alongside capacity constraints, duplicate registrations further inflated the workload and obscured the true number of unique patients awaiting care. Staff believed that *“a large portion of the nine hundred are duplicates,” (S2)* driven by lack of acknowledgment messages, repeated patient attempts, and the ease of link sharing.

#### 3.2.5. Theme 5: Digital Literacy, Information Quality, and Platform Preferences

Both groups demonstrated high general digital literacy; however, staff emphasized that comfort with technology did not equate to clinically useful data.

##### Subtheme 1: High Digital Confidence and Institutional Trust

The digital experience was commonly framed as modern and convenient, reinforcing positive perceptions of the system’s usability. Some patients preferred the web-based link because they viewed it as more dependable, explaining that *“Honestly, the link is better. Applications can sometimes be new and have technical issues, but websites are usually stable and reliable” (P13)*.

Patients also expressed strong trust in a university’s digital infrastructure. As one patient explained, *“I think the digital platform is better more secure and trustworthy especially since it’s from Umm Al-Qura itself” (P1)*. For many, institutional credibility increased their confidence in using and relying on the digital triage system.

In contrast, staff cautioned that general digital comfort does not ensure equitable access. One staff noted that *“some patients cannot read or write and come with someone who enters the information for them, sometimes incorrectly” (S1)*, highlighting that surface-level digital readiness can mask vulnerabilities that require additional support or alternative pathways.

##### Subtheme 2: Health-Specific Literacy Gaps and Information Quality

Despite patients’ comfort with the digital platform, staff emphasized that many submissions lacked the clinical detail required for accurate triage. This gap was attributed less to the technology itself and more to limitations in health-specific literacy, including difficulties describing symptoms, distinguishing between treatment needs, and capturing relevant clinical images. As one faculty member explained, *“The patient cannot differentiate between endodontic and operative cases even pain is not an indicator; it could be an abscess or a necrotic tooth” (F1)*.

Photographs and supporting documents were consistently described as essential for remote assessment, yet their usefulness depended heavily on patient understanding. Faculty noted that many images were unclear or incomplete, limiting diagnostic value. One faculty explained that patients often submit photos *“but not the right tooth, not the right angle, or without good lighting” (F2)*.

#### 3.2.6. Theme 6: Improvement Pathways and Teledentistry Potential

Across both patient and staff accounts, participants articulated clear and largely complementary priorities for improving the digital triage system and expanding teledentistry functionality. While patients emphasized communication and responsiveness, staff focused on structural redesign, integration, and automation.

##### Subtheme 1: Communication as the Primary Patient Priority

Patients consistently identified communication as the most urgent area for improvement. They emphasized the need for immediate confirmation, regular status updates, clearer timelines, and timely appointment notifications. Even when patients viewed the initial registration process positively, the absence of follow-up communication undermined confidence in the system. As one participant stated, *“We need faster responses or processing requests more quickly” (P16)*. Although some patients acknowledged high demand and system pressure, they stressed that predictable updates rather than speed alone were essential to reducing uncertainty and maintaining trust.

##### Subtheme 2: Redesigning the Triage Form for Clarity and Clinical Relevance

Staff proposed targeted changes to improve the accuracy and usefulness of information captured through the triage form. They emphasized translating clinical terminology into patient-friendly language and adopting more structured formats, such as checklists and guided questions. As one faculty member noted, *“Patients of lower education will say I have a hole or cavity, they may not understand technical terminology” (F5)*.

##### Subtheme 3: Integration, Automation, and Analytics to Support Workflow Continuity

Beyond form design and communication, staff highlighted system integration as essential for long-term sustainability. Desired features included interoperability with electronic health records, appointment scheduling, national identity verification, and automated reporting. As one director explained, *“Having weekly statistics how many patients entered, how many were treated would help improve transparency and reduce complaints” (S1)*.

Patients also expressed strong interest in expanding the system into a dedicated mobile application, associating it with transparency, security, and real-time communication.

### 3.3. Comparison of Patient and Staff Perspectives Across Themes

To further explore similarities and differences between stakeholder groups, a cross-stakeholder comparison was conducted. While both patients and staff shared concerns regarding communication gaps, delays, and system inefficiencies, their perspectives diverged in relation to clinical accuracy, workflow constraints, and expectations of care. A summary of convergence and divergence across the main themes is presented in Table 3.

## 4. Discussion

This study examines the use of a digital dental triage system within an academic dental setting and identifies a clear disconnect between what patients expect and how the system currently functions. While patients consistently described the system as easy to use, this sense of usability did not translate into overall satisfaction.

Some subthemes appeared more prominently in staff accounts than in patient narratives, particularly those related to clinical processes, workflow management, and institutional constraints. This likely reflects the differing roles of the two groups, as staff have greater insight into system-level operations that may not be visible to patients. Rather than seeking equal representation across groups, the analysis focused on capturing the distinct perspectives each group brought to the study. These differences were treated as analytically meaningful, offering complementary insights into how the digital triage system operates.

Gaps in communication, delayed responses, and unclear care pathways undermined confidence and limited the perceived value of the triage process. These findings suggest that, from the patient’s perspective, system success is defined not only by how simple it is to complete, but by whether it provides timely feedback, clear direction, and a meaningful pathway to care [19,26]. At the same time, staff tended to define system quality in terms of clinical adequacy and diagnostic usefulness, highlighting a clear difference in how success was understood by users and providers. This divergence in criteria contributed to frequent misclassification, inefficient patient routing, and recurring referral loops, illustrating the limitations of relying on simplified, unstructured self-reported data for clinical decision-making in digital triage systems [6,14]. Rather than reflecting individual error, these challenges point to structural tensions between patient-friendly design and the clinical detail required for effective triage.

The observed usability–satisfaction gap can be understood through Expectation-Confirmation Theory, which suggests that satisfaction depends largely on whether system performance aligns with users’ prior expectations of outcomes [27,28]. In this study, patients often associated completion of the triage form with prompt communication and appointment scheduling. When these expectations were not met, satisfaction declined even though the system itself was perceived as easy to use. These findings are consistent with broader digital health literature showing that usability alone is insufficient to sustain engagement or satisfaction in the absence of meaningful feedback, transparency, and perceived responsiveness [19,27,28]. Similarly, research on mHealth interventions suggests that patient satisfaction is shaped more by perceived effectiveness, communication quality, and continuity of care than by interface simplicity alone [19,29]. Taken together, the mismatch observed in this study reflects a breakdown in feedback mechanisms and expectation management, rather than a failure of interface design itself.

While Expectation Confirmation Theory helps explain how patients evaluate system performance against their expectations, the findings also highlight broader system-level influences. Factors such as workflow integration, communication processes, institutional constraints, and staff capacity shaped how digital triage functioned in practice. The gap between patient expectations and system delivery therefore reflects not only a perceptual mismatch but also an implementation challenge within a complex healthcare setting. Considering both perspectives provides a more comprehensive understanding of digital triage performance.

Communication breakdown emerged as the most critical system-level weakness, directly linking patient dissatisfaction with inefficiencies in care delivery. From a dental public health perspective, the absence of post-registration communication such as confirmation, status updates, or clear next steps creates barriers to access, particularly for patients with urgent or time-sensitive oral health needs. This finding is consistent with previous studies, such as Sexton et al. (2022), which identified communication as a key determinant of patient experience in digital triage systems [6], and Woods et al. (2019), who reported that unclear communication pathways negatively affect user engagement in mHealth platforms [19]. Patients’ descriptions of the system as a “black box” highlight how silence after submission led to uncertainty, repeated registrations, prolonged waiting, and loss of trust. Similar patterns have been reported in telehealth research, where inadequate communication is associated with increased anxiety, reduced engagement, and poorer perceived quality of care [6,19,30].

These communication failures also carry implications for equity and unmet need. Delays, unclear pathways, and repeated triage attempts disproportionately affect patients with lower health literacy, limited digital access, or fewer resources to navigate the system, potentially exacerbating existing oral health disparities. Evidence from digital triage studies indicates that weak feedback loops can compromise both patient safety and continuity of care, particularly when triage outputs are poorly coordinated with downstream services [6,29]. Staff accounts in this study further suggest that communication gaps operate across multiple levels, patient–system, staff–staff, and system-level governance, underscoring the need for standardized protocols and transparent reporting.

The findings also reveal a policy-relevant tension between simplicity and clinical adequacy. While simplified triage forms improve accessibility and uptake, over-reliance on unstructured patient self-reporting can undermine diagnostic accuracy and efficient resource allocation. This aligns with previous research, such as Dornellas et al. (2023) and Liu et al. (2024), which highlight the importance of structured data capture and the limitations of relying solely on patient-reported information for accurate clinical triage [12,18]. From a public health standpoint, strengthening communication, standardization, and system integration is essential to ensure that digital triage supports equitable access, minimizes unmet oral health needs, and contributes to sustainable service delivery in publicly funded and academic dental settings.

This study contributes to dental public health by highlighting how organizational and educational structures shape the real-world performance of digital triage systems in teaching hospitals. In academic dental settings, triage decisions are inherently influenced by student competency levels, case allocation requirements, and limited clinical capacity. Patients, however, are largely unaware of these constraints. As a result, delays, non-response, or case reassignment are often interpreted as system inefficiencies rather than as reflections of institutional realities. These findings underscore the importance of evaluating digital health interventions within their operational and educational contexts, particularly where care delivery and workforce training are closely intertwined.

Both patients and staff identified capacity limitations and workflow inefficiencies as major barriers to system effectiveness. From the patient perspective, these constraints manifested as prolonged and uncertain waiting periods, while staff described increased administrative burden due to duplicate registrations, unclear prioritization, and reduced clinical efficiency. These findings are consistent with previous research, such as Alrumaim et al. (2025) and Riboli-Sasco et al. (2023), which reported that digital triage systems can increase demand without a corresponding expansion in clinical capacity, particularly when communication pathways and feedback mechanisms are limited [31,32]. In this study, repeated registrations were closely linked to the absence of confirmation or updates, highlighting how communication failures can inflate demand, obscure unmet need, and place additional strain on already limited public dental resources.

The suggested improvements point away from isolated usability enhancements and toward system-level redesign grounded in public health principles. Patients emphasized the need for consistent communication such as confirmation messages, real-time status updates, and transparent waiting times to transform uncertainty into informed waiting and restore trust. Staff, in turn, focused on improving input quality and workflow efficiency through simpler language, structured symptom checklists, and guided image capture to reduce misclassification. Both groups converged on the importance of stronger digital integration, including interoperability with electronic health records, automated detection of duplicate registrations, and real-time reporting. Together, these recommendations reflect the need to align patient-facing interfaces with back-end system intelligence to support equitable access, efficient resource allocation, and sustainable service delivery [7,33].

The implications of this study extend to the design and implementation of next-generation digital triage systems in dentistry. Emerging technological tools offer potential solutions to many of the challenges identified. For example, AI-assisted symptom checkers and image-based diagnostic tools, such as recently developed mobile health (mHealth) applications for detecting oral conditions, could enhance the accuracy of patient-reported information and reduce misclassification [34]. Tools incorporating guided image capture and automated clinical assessment may support more reliable remote triage, particularly for conditions that are difficult for patients to describe using text alone.

In addition, real-time communication platforms and automated notification systems could address the communication gaps identified in this study by providing timely updates and clearer care pathways. Integration with electronic health records and the use of predictive analytics may further improve workflow efficiency and patient routing. Together, these technologies highlight how combining digital usability with clinically informed decision support systems may strengthen both patient experience and system performance in academic and public dental healthcare settings.

This study contributes to the limited qualitative literature on digital dental triage in academic settings by incorporating both patient and staff perspectives and by examining system-level factors beyond usability alone [35]. The findings offer practical insights for academic and public dental services facing similar capacity pressures. Teaching hospitals often share key characteristics, including the integration of clinical care and student training, which can give rise to comparable challenges related to triage, workflow, and patient expectations. At the same time, differences in organizational structure, patient populations, and digital infrastructure across institutions may shape how these systems function in practice.

The findings are particularly relevant to publicly funded dental systems managing high demand and constrained resources. However, differences in health system organization, workforce capacity, and levels of digital adoption should be considered when applying these results to other settings. Future multi-site studies would help assess how digital triage systems perform across a wider range of academic and public healthcare contexts.

This study did not specifically examine differences in experiences across patient characteristics such as age, education, or digital literacy. As a result, potential variation between subgroups may not have been fully captured. Future research could explore how these factors influence patient engagement with digital triage systems.

In addition, interviews were conducted using different modes (telephone for patients and in person for staff), which may have influenced the depth or nature of responses. While steps were taken to maintain consistency across interview formats, these differences should be considered when interpreting the findings.

## 5. Conclusions

This study shows that the success of digital dental triage systems depends on more than ease of use alone. While usability encourages uptake, effective triage in academic dental settings also requires clear communication, timely feedback, and clinically meaningful pathways that align with institutional capacity and educational mandates. From a dental public health perspective, these findings highlight the importance of transparency, system integration, and expectation management in ensuring equitable access to care and efficient use of limited resources. Strengthening these elements can improve patient experience, reduce unnecessary demand and duplication, and support sustainable service delivery, particularly within teaching hospitals that serve diverse and high-need populations.

## Figures and Tables

**Table 1:** Topic guide.

Patient Interview Topic Guide	Staff and Faculty Interview Topic Guide
Opening/Context • Can you tell me a little about what brought you to the dental hospital today?	• Could you tell me about your role at the dental teaching hospital and how it relates to patient triage or clinic allocation?
Experience with the Electronic Triage System • Can you tell me the story of how you came to use the digital triage link? • Before using the link, what did you expect it would help you with? • Can you walk me through your experience of completing the triage form, from start to finish? • How did you find the process of explaining your dental problem using the form?	• Can you describe your experience with the current digital triage system? • How would you describe the overall purpose of the digital triage system from your perspective? • How does the triage system currently work in practice, from your point of view? • What do you see as the main strengths of the digital triage system? • What challenges have you observed in how the system functions?
Perceived Outcomes and Follow-Up • After submitting the triage form, what happened next? • How did you feel about the communication or feedback you received after completing the form? • How did this process affect your understanding of what would happen next in your care?	• Can you describe how triage decisions are made once patients submit their information? • How does the system support or limit accurate clinical classification and case routing? • How do educational requirements (e.g., student level, competency, quotas) shape triage and case allocation decisions?
Expectations and Meaning • Looking back, how well did the triage process meet your expectations? • How did the experience make you feel about the hospital and its services more generally? • In what ways, if any, did the triage process influence how confident you felt about getting the care you needed?	• How do you think patients understand the triage process and what it can realistically deliver? • What mismatches, if any, do you see between patient expectations and institutional capacity? • How do these mismatches affect patient satisfaction and staff workload?
Suggestions for Improvement and Closing • From your perspective, what changes would make the triage system work better for patients? • Is there anything else about your experience with the digital triage system that you think is important for us to understand? • Is there something you wish you had been asked, but weren’t?	• From your perspective, what changes would most improve the effectiveness of the digital triage system? • How could the triage form be redesigned to better balance patient usability and clinical accuracy? • How do you see the role of automation, system integration, or analytics in improving triage and clinic operations?

**Table 2:** Themes and categories identified through thematic analysis.

Themes	Subthemes
1. Interface usability versus clinical needs	- Patients prioritize usability and accessibility - Staff concerns about insufficient clinical detail and misclassification
2. Communication breakdown as the core system failure	- Post-registration silence for patients - Internal coordination gaps and public complaint exposure
3. Hidden organizational and educational constraints	- Educational matching is necessary but invisible - Patient expectations of universal eligibility - Structurally determined dissatisfaction
4. Operational consequences of capacity mismatch	- Workflow bottlenecks consume clinical time - Demand-supply imbalance and duplicate registrations
5. Digital literacy, information quality, and platform preferences	- High digital confidence and institutional trust - Health-specific literacy gaps and information quality
6. Improvement pathways and teledentistry potential	- Communication as the primary patient priority - Redesigning the triage form for clarity and clinical relevance - Integration, automation, and analytics to support workflow continuity

**Table 3:** Convergence and divergence in patient and staff perspectives across themes.

Themes	Areas of Convergence	Areas of Divergence
1. Interface usability versus clinical needs	Both groups recognized the system as accessible and easy to use	Patients valued simplicity; staff viewed it as lacking clinical detail
2. Communication breakdown as the core system failure	Both identified communication gaps as a major issue	Patients experienced silence; staff focused on coordination challenges
3. Hidden organizational and educational constraints	Both acknowledged delays and system constraints	Patients expected immediate care; staff emphasized educational limitations
4. Operational consequences of capacity mismatch	Both reported inefficiencies and delays	Patients perceived waiting and uncertainty; staff highlighted duplication and workload burden
5. Digital literacy, information quality, and platform preferences	Both valued digital access	Patients confident in use; staff emphasized data quality limitations
6. Improvement pathways and teledentistry potential	Both supported system improvement	Patients prioritized communication; staff prioritized redesign and integration

## Data Availability

The raw data supporting the conclusions of this article will be made available by the authors upon request.

## Supplementary Materials

The following supporting information can be downloaded at: https://www.mdpi.com/article/10.3390/dj14070423/s1, File S1: COREQ checklist [36].

## References

[1] Sharka R., Sedayo L., Morad M., Abuljadayel J. (2024). Measuring the impact of dental service quality on revisit intention using an extended SERVQUAL model. Front. Oral Health.

[2] Amin S., Zaheer K., De Souza M. (2021). Dental public health in action: Utilising a telephone triage system to run an urgent dental care hub during the COVID-19 pandemic. Community Dent. Health.

[3] Beauquis J., Petit A.E., Michaux V., Sagué V., Henrard S., Leprince J.G. (2021). Dental emergencies management during the COVID-19 pandemic peak: A cohort study. J. Dent. Res..

[4] Ali S.A., Al-Qahtani A.M.A., Al Banai S.R., Albaker F.J., Almarri A.E., Al-Haithami K., Khandakji M.N., El Ansari W. (2022). Role of newly introduced teledentistry service in the management of dental emergencies during the COVID-19 pandemic in Qatar: A cross-sectional analysis. Telemed. J. e-Health.

[5] Park H., Shin D.S., Wade C., Duong D.B.L. (2025). Scoping review of nurse triage in primary care. BMC Nurs..

[6] Sexton V., Dale J., Bryce C., Barry J., Sellers E., Atherton H. (2022). Service use, clinical outcomes and user experience associated with urgent care services that use telephone-based digital triage: A systematic review. BMJ Open.

[7] Kengne Talla P., Allison P., Bussières A., Rodrigues A., Bergeron F., Giraudeau N., Emami E. (2025). Teledentistry for improving access to and quality of oral health care: Overview of systematic reviews and meta-analyses. J. Med. Internet Res..

[8] Park H.N., Lee K., Wallace C. (2026). The use of triage in primary care in the UK: An integrative review and narrative synthesis. J. Adv. Nurs..

[9] Farzandipour M., Nabovati E., Sharif R. (2024). The effectiveness of tele-triage during the COVID-19 pandemic: A systematic review and narrative synthesis. J. Telemed. Telecare.

[10] Gurgel-Juarez N., Torres-Pereira C., Haddad A.E., Sheehy L., Finestone H., Mallet K., Wiseman M., Hour K., Flowers H.L. (2022). Accuracy and effectiveness of teledentistry: A systematic review of systematic reviews. Evid. Based Dent..

[11] Yang L., Pang J., Zuo S., Xu J., Jin W., Zuo F., Xue K., Xiao Z., Peng X., Xu J. (2024). Evolution of the “Internet Plus Health Care” mode enabled by artificial intelligence: Development and application of an outpatient triage system. J. Med. Internet Res..

[12] Dornellas A.P., Marques J.V., Aníbal Oliveira dos Santos I., Ramos M., Mulder J., Haddad A.E. (2023). A novel questionnaire to perform tele-triage of dental emergencies in children: A before-and-after study nested within a randomized clinical trial. F1000Research.

[13] Boggan J.C., Shoup J.P., Whited J.D., Van Voorhees E., Gordon A.M., Rushton S., Lewinski A.A., Tabriz A.A., Adam S., Fulton J. (2020). Effectiveness of acute care remote triage systems: A systematic review. J. Gen. Intern. Med..

[14] Kopka M., von Kalckreuth N., Feufel M.A. (2025). Accuracy of online symptom assessment applications, large language models, and laypeople for self-triage decisions. npj Digit. Med..

[15] Alatawi A.D., Niessen L.W., Khan J.A.M. (2020). Efficiency evaluation of public hospitals in Saudi Arabia: An application of data envelopment analysis. BMJ Open.

[16] Al-Anezi F.M. (2025). Challenges of healthcare systems in Saudi Arabia in delivering Vision 2030: An empirical study from healthcare workers’ perspectives. J. Healthc. Leadersh..

[17] Nguyen H., Meczner A., Burslam-Dawe K., Hayhoe B. (2022). Triage errors in primary and pre-primary care. J. Med. Internet Res..

[18] Liu V., Kaila M., Koskela T. (2024). Triage accuracy and the safety of user-initiated symptom assessment with an electronic symptom checker in a real-life setting: Instrument validation study. JMIR Hum. Factors.

[19] Woods L.S., Duff J., Roehrer E., Walker K., Cummings E. (2019). Patients’ experiences of using a consumer mHealth app for self-management of heart failure: A mixed-methods study. JMIR Hum. Factors.

[20] Thobaity A.A. (2025). Identifying the main bottlenecks in the workflow of Saudi Arabian emergency departments. J. Nurs. Manag..

[21] Abdul Majid M.A., Othman M., Mohamad S.F., Lim S., Yusof A. (2017). Piloting for interviews in qualitative research: Operationalization and lessons learnt. Int. J. Acad. Res. Bus. Soc. Sci..

[22] Braun V., Clarke V. (2006). Using thematic analysis in psychology. Qual. Res. Psychol..

[23] Birt L., Scott S., Cavers D., Campbell C., Walter F. (2016). Member checking: A tool to enhance trustworthiness or merely a nod to validation?. Qual. Health Res..

[24] Saunders B., Sim J., Kingstone T., Baker S., Waterfield J., Bartlam B., Burroughs H., Jinks C. (2018). Saturation in qualitative research: Exploring its conceptualization and operationalization. Qual. Quant..

[25] Morse J.M. (2015). Critical analysis of strategies for determining rigor in qualitative inquiry. Qual. Health Res..

[26] Wijaya S.S., Afifi S. (2024). Exploring user satisfaction in digital healthcare: The role of doctor–patient communication, service quality, and user experience on Halodoc’s online medical platform. Commun. Humanit. Soc. Sci..

[27] Zhang Y., Wu P. (2024). Continuous adoption of online healthcare platforms: An extension of the expectation-confirmation model and network externalities. BMC Public Health.

[28] Ma X., Li Y., Suo A. (2025). Revealing the dynamics of mobile health service continuance intention: Effects of expectation, confirmation, and chronic disease. Front. Public Health.

[29] Inal Y., Wake J.D., Guribye F., Nordgreen T. (2020). Usability evaluations of mobile mental health technologies: Systematic review. J. Med. Internet Res..

[30] Vitale R., Smith S., Doolittle B.R. (2019). Improving patient satisfaction through improved telephone triage in a primary care practice. Fam. Med. Community Health.

[31] Alrumaim S., Farsi N., Farsi N., Baghlaf K., Farsi D. (2025). Feasibility and acceptability of pediatric teledentistry: A mixed-methods study. Int. J. Paediatr. Dent..

[32] Riboli-Sasco E., El-Osta A., Alaa A., Webber I., Karki M., Asmar M.L.E., Purohit K., Painter A., Hayhoe B. (2023). Triage and diagnostic accuracy of online symptom checkers: Systematic review. J. Med. Internet Res..

[33] Rouidi M., Elouadi A.E., Hamdoune A., Choujtani K., Chati A. (2022). TAM-UTAUT and the acceptance of remote healthcare technologies by healthcare professionals: A systematic review. Inform. Med. Unlocked.

[34] Chau R.C.W., Gong Z., Cheng A.C.C., Mao K., Thu K.M., Zhang R., Ling Z., Chang T.H., Tan H.J., Tsang H.S.-H. (2026). External validation of a mHealth tool for detecting gingival inflammation in community-dwelling older adults. J. Dent..

[35] Irving M., Stewart R., Spallek H., Blinkhorn A. (2018). Using teledentistry in clinical practice as an enabler to improve access to clinical care: A qualitative systematic review. J. Telemed. Telecare.

[36] Tong A., Sainsbury P., Craig J. (2007). Consolidated criteria for reporting qualitative research (COREQ): A 32-item checklist for interviews and focus groups. Int. J. Qual. Health Care.

